# The development of national entrustable professional activities to inform the training and assessment of public health and preventative medicine residents

**Published:** 2017-06-30

**Authors:** Brent Moloughney, Kieran Moore, Damon Dagnone, David Strong

**Affiliations:** 1 Dalla Lana School of Public Health, University of Toronto, Ontario, Canada; 2 Queen’s University, Ontario, Canada; 3 Department of Emergency Medicine, Queen’s University; 4 Department of Community Health Sciences, University of Calgary, Alberta, Canada

## Abstract

**Background:**

Entrustable Professional Activities (EPAs) have emerged to bridge the gap between the learning of individual competencies and competence in real world practice. EPAs capture the critical core work of a discipline integrating competencies from multiple domains. This report describes the development of a set of EPAs for specialty training in Public Health and Preventive Medicine (PHPM) in Canada.

**Methods:**

The PHPM EPAs were developed using multiple existing sources. A combination of workshops and a national online survey was used to consult with PHPM program directors, the national specialty committee, and competency-based education experts.

**Results:**

A national survey of PHPM program directors had a 71% response rate with 80% or more of respondents agreeing with all of the 20 EPA titles and all but one of their descriptions. Competency developmental stage-specific milestones were identified for each EPA.

**Conclusion:**

The identification of the EPAs and their milestones will increase emphasis on the demonstrated performance of the specialty’s core work. Simulations applicable to several EPAs have been developed. The EPAs have also been incorporated into a PHPM National Review Course and will be used to develop a national PHPM curriculum, as well as a national written practice examination.

## Introduction

Objectives of training provide detailed lists of competencies to be acquired in post-graduate training.^1^ While competencies provide a blueprint for curricula, hundreds of competencies of varying granularity are challenging to translate for training and assessment in practice-based settings.^2^ Over the past decade, the concept of Entrustable Professional Activities (EPAs) has emerged to bridge the gap between the learning of individual competencies and competence in real world practice.^3^

**Key Concepts**
**Competency-Based Medical Education (CBME): “**an outcomes-based approach to the design, implementation, assessment, and evaluation of medical education programs, using an organizing framework of competencies.”^4^
**Competency:** “an observable ability of a health professional, integrating multiple components such as knowledge, skills, values, and attitudes.”^4^
**Entrustable professional activity (EPA):** “a key task of a discipline that can be entrusted to an individual who possesses the appropriate level of competencies.”^5^
**Milestone: “**the expected ability of a health care professional at a stage of expertise.”^5^

Entrustable Professional Activities are units of work that together “constitute the mass of critical elements that operationally define a profession.”^6^ Typically numbering between 20–30 EPAs per discipline,^7^ EPAs identify what a professional does in practice and how to know a graduate is competent to independently and safely practice these activities.^3^ Milestones complement EPAs by identifying the expected ability of a health professional for achieving an EPA by a particular stage of development.^5^ Fulfilling a milestone requires the application of multiple competencies.

A growing number of medical disciplines in Canada and elsewhere have begun developing and applying EPAs in their training programs including family medicine, geriatrics, and internal medicine.^8^–^11^ Entrustable Professional Activities have not previously been applied to the training of public health physicians, whose focus is on protecting and promoting the health of populations.

In Canada, public health and preventive medicine (PHPM) specialists train in a 5-year, post-MD Royal College of Physicians and Surgeons (RCPSC) residency program comprised of: 1–2 years of clinical training; at least two (4-month) semesters of academic public health training; and, at least 18 months of supervised placements in public health-related practice settings.^12^ Graduates work at local (e.g., Medical Officer of Health), provincial-territorial, federal and international system levels, as well as academic, clinical, and private sector settings.

With a recently released set of detailed PHPM objectives of training,^1^ EPAs offer to translate these into a coherent set of practice-based outcomes and provide a consistent focus on residents’ development throughout the various stages of PHPM training. This is particularly relevant since PHPM residents have had one of the lowest final examination success rates of the RCPSC specialties.^13^

With Queen’s University School of Medicine setting the goal for its postgraduate specialty programs to adopt CBME for all incoming residents, an opportunity to pursue EPA development for PHPM was created. As a small specialty, collaboration was sought with other interested PHPM programs, initially in Ontario and then nationally. Representatives from two other PHPM programs joined the Ontario programs to lead this project with the remaining programs providing feedback on the developed EPAs.

While the development of PHPM EPAs is program-driven, this work aligns with the Royal College’s *Competence by Design* (CBD) initiative, which is transforming resident education to be more outcome-focussed with, among other features, the identification of training stage-specific EPAs and milestones.^14^ With PHPM’s participation in this initiative scheduled to commence in 2019,^15^ the Royal College indicated to the National Specialty Committee that the experience defining and applying EPAs would be useful for informing the specialty’s future participation in the CBD initiative. While EPAs are conceptually attractive, their application poses a number of challenges to programs, residents and faculty including mechanisms and tools to assess the achievement of EPAs.^7^

The purpose of this report is to describe the development of a set of end-of-training EPAs for PHPM. This specialty’s approach to, and experience in, developing EPAs will be informative to other disciplines considering a similar undertaking.

## Methods

**Figure 1:**
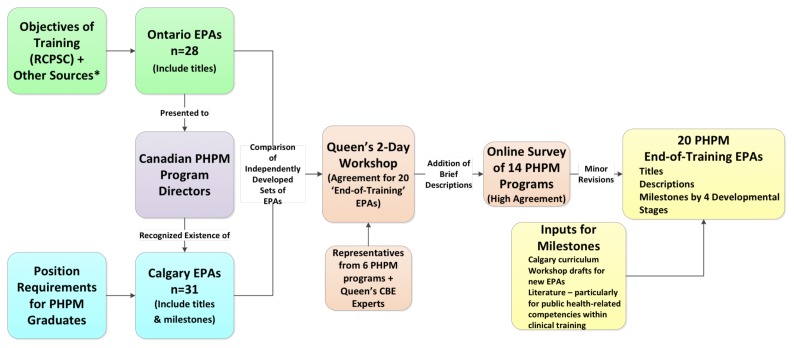
Key process steps in development of PHPM EPAs *Other sources used included public health physician competencies from Canada, the U.S., United Kingdom, and Australia/New Zealand.

The EPA development process is illustrated in Figure 1. With the support of Queen’s University, the Ontario-based programs developed a draft set of EPA titles utilizing multiple resources including the minimum competencies for Medical Officers of Health,^16^ the development of which had involved practitioners from across Canada. In sharing the draft EPAs among PHPM programs nationally, it became known that the University of Calgary PHPM program had developed a set of EPAs based on PHPM position descriptions in that province.

With two independently developed sets of PHPM EPAs, created through different methods, it was decided to reconcile them into a single set and to then seek feedback on it from all PHPM programs. If there were low levels of acceptance of the EPAs or critical gaps identified, then a subsequent Delphi process would be pursued. To prepare the PHPM EPAs, Queen’s University hosted a two-day workshop attended by interested program directors or their representatives from six of the 14 PHPM programs, as well as competency-based education experts and a public health workforce development consultant.

Working with the combined total of 59 EPA titles, workshop participants consolidated them into 20 final EPAs of which six were new. Workshop participants began development of descriptions and milestones for these new EPAs with post-workshop further development to ensure descriptions addressed EPAs’ scope and limitations.^7^

An online survey was conducted of all PHPM program directors across Canada. Their level of agreement with each of the EPA titles and descriptions was assessed using a five-point scale with the anchors “strongly agree,” “agree,” “neither agree nor disagree,” “disagree,” and “strongly disagree.” Additional comments or suggestions for improvement were also sought for each item.

The survey results were discussed by the group of program directors and external experts that had attended the workshop and minor revisions were made to the EPA titles and descriptions. This was deemed sufficient in order for the EPAs to be piloted while awaiting the Royal College’s CBD process.

To address the milestones that would accompany the EPAs, the CanMEDS 2015 competency developmental stages were aligned with the four stages of PHPM training (Table 1). While the original 31 Calgary PHPM EPAs had accompanying milestones, these needed to be resorted to align with the smaller number of 20 final EPAs. For newly identified EPAs, workshop participants began development of milestone statements, which were completed by the project consultant. An additional gap in milestones was created when many of Calgary’s existing “transition to discipline” milestones were shifted to later developmental stages where they would be more realistic to achieve for most programs. Multiple sources were utilized to identify public health-related competencies that could be reasonably acquired within clinical training settings during the “transition to discipline” developmental stage.^16^–^18^

This EPA project was deemed to be exempt from a formal ethics review by the Chair of the University of Queen’s Research Ethics Board since those involved were not viewed as research participants.

## Results

Half of the final 20 EPA titles were easily identified from the comparison of the two pre-existing EPA sets from the University of Calgary and the Ontario-based PHPM programs. To resolve the remaining practice areas, a key decision was made to focus on EPAs reflecting the core tasks achieved by the end-of-training. “Overarching”-type EPAs were utilized to encompass multiple more granular, “nested EPAs,”^2^ which would all need to be demonstrated in order to successfully complete the overarching EPA (Table 2). While termed “end-of-training,” it was explicitly recognized that a resident could demonstrate earlier fulfilment of an EPA since the demonstration of competence is not always dependent upon a specific amount of training time.^19^

The survey of program directors achieved a 71% response rate with a total of 11 respondents from 10 of the 14 PHPM. One of the respondents did not complete the survey, but had attended the two-day workshop and indicated agreement with the EPA titles and descriptions. Combining results of “strongly agree” and “agree” categories from the 5-point scale, 80% or more of survey respondents agreed with all of the EPA titles and all but one of their descriptions (Table 3).

The survey results were used to make minor improvements to the EPA titles and descriptions with particular attention to the distinctions and inter-dependencies between EPAs (see Appendix A for an example).

The milestones were reviewed to assess their alignment with the finalized EPA titles and descriptions with improvements made where necessary; Table 4 provides an example of the final EPA for managing a communicable disease outbreak (see Table 4 in Appendix B).

## Discussion

The purpose of this report is to describe the development of a set of end-of-training EPAs to capture the core, critical tasks of PHPM practice. This specialty’s approach to, and experience in, developing EPAs will be informative to other disciplines considering a similar undertaking. Consistent with guidelines for EPA development,^7^ a combination of expert meetings and surveys were utilized to consolidate and refine previously developed sets of EPA titles. The final EPA titles and descriptions received high levels of agreement among PHPM program directors across the country, otherwise a Delphi approach would have been required.

The EPAs are anticipated to be informative for residents, residency programs and faculty members. For residents, the EPAs provide an explicit description of the core tasks of the specialty by identifying, through milestones, the expected ability for achieving an EPA at each stage of development (see Table 4 in Appendix B for example). This will support residents to be more proactively engaged in their training to ensure that they are getting the necessary experiences and are being observed and assessed.^7^

Greater stage-specific clarity will benefit PHPM programs since they are reliant on clinical training programs and graduate academic programs to provide their residents with public health-related competencies associated with the care of individual patients, as well as graduate-level public health-related knowledge and skills, respectively. These are foundational to the PHPM-specific training rotations that occur in the final years of the residency program. In these later training stages, the EPAs will support and encourage a greater emphasis on observing residents demonstrating their competence for specific, relevant tasks. This is a further step from historical medical training perspectives that assumed a defined time period within a placement would result in sufficient acquisition of needed competencies.^19^ Instead, while time is viewed as a resource for acquiring competencies, the focus of training is outcome-oriented with the demonstrated achievement of the milestones and EPAs.^20^

Implementation of EPAs requires committed, experienced and highly-trained faculty.^2^ While providing clearer guidance on the continuum of learning and milestones for assessing development, EPAs place greater demands for making formal assessment and promotion decisions, establishing portfolio systems to capture and track assessments, and to train and mentor faculty.^7^ For example, a family medicine program created electronic EPA field notes to structure day-to-day assessment and feedback, and to serve as a summative tool to ground competency declarations about residents.^8^ In the absence of mechanisms and tools to assess the achievement of EPAs, they risk being another competency-related conceptual framework collecting dust.

Some EPAs such as response to a public health emergency may not be universally observable for all residents. Nevertheless, residents need to be prepared for these scenarios, which is why they are included in the EPAs. The implication is that programs require a variety of tools (simulations among them) which can be used to provide residents with exposure and practice opportunities.

The Queen’s PHPM program has developed simulations that are directly applicable to several EPAs including public health emergencies, outbreak management, and cancer cluster investigation, which will be made available to residents in all programs, as well as a refresher to existing PHPM physicians. Additional case-based learning tools and simulations will be needed to support and assess residents’ development. Considering the small size of each PHPM program, a national approach to development should be pursued if possible.

The EPAs have already been incorporated into the first annual PHPM National Review Course.^21^ There has also been agreement among program directors to use the EPAs in developing a national PHPM curriculum and a national written practice examination. Several PHPM programs have started or are looking to pilot the EPAs on a voluntary basis and their experience will be informative when the specialty formally enters the RCPSC’s CBD process to integrate EPAs and milestones across the curriculum.^22^

Overall, it is anticipated that the development of EPAs will strengthen the recruitment, training and assessment of PHPM residents, which will collectively lead to stronger PHPM practice. Greater standardization of competence for agreed-upon core tasks will support greater employer confidence for what can be expected of a PHPM graduate. In addition to better tracking of residents’ training progress, monitoring the success rate of PHPM residents in their final RCPSC examination are among potential impact measures.

### Conclusion

Entrustable Professional Activities are a vital bridge between detailed lists of learning objectives and the training and assessment of the core, critical tasks of a specialist. A set of 20 end-of-training EPAs have been identified for PHPM, which are anticipated to strengthen the recruitment, training and assessment of future PHPM specialists. Implementation of the EPAs will have wide-spread implications for residents, residency programs and teaching faculty.

## Figures and Tables

**Table 1:** Alignment of PHPM training components with CanMEDS competency development stage

PHPM Training Component	CanMEDS 2015 Developmental Stage
1–2 years of post-MD clinical training	Transition to discipline
2+ (4 month) semesters of academic public health courses	Foundations of discipline
Preliminary ‘core’ practicum rotations	Core of discipline
Final months of residency	Transition to practice

**Table 2:** List of overarching-type EPAs and their constituent nested EPAs

Overarching-Type EPA	Nested EPAs
Manage communicable diseases of public health importance	Manage an animal bite exposure Manage a report of a communicable disease Manage a blood and body fluid exposure Manage an infection prevention and control break (community) Manage a reported case of a sexually transmitted infection Manage a reported case of active or latent tuberculosis Manage an adverse report of an event following immunization Provide behind-schedule or off-schedule immunization recommendations
Manage environmental health risks, exposures and incidents	Manage an air quality issue Manage a drinking or recreational water issue Manage a food safety issue
Lead and manage strategic planning	Business case development
Lead and manage operational planning and evaluation of a public health program	Operational planning Program evaluation
Lead and manage a team and organization	Develop and manage a budget Manage a project Manage/mediate a conflict Hire, performance manage and discharge staff

**Table 3:** Levels of program directors’ agreement with the draft EPAs and descriptions (n=10)

EPAs*	% Agree with	
	EPA Title	EPA Description
**Domain: Monitor and assess the health of the public**		
Conduct a population health status assessment	100%	92%
Conduct a health needs assessment	100%	83%
Design and manage health surveillance systems	91%	82%
Conduct a health impact assessment	91%	90%
Conduct an environmental health risk assessment	100%	91%
**Domain: Public Health Leadership & Management**		
Act as spokesperson to communicate about public health issues to the public, their elected representatives, inter-sectoral partners, and the health system.	100%	80%
Lead and manage strategic planning	82%	73%
Lead and manage the operational planning and evaluation of a public health program	100%	90%
Lead and manage a team and organization.	90%	100%
Lead and manage change within an organization	100%	89%
Lead and manage a quality improvement initiative	80%	100%
**Domain: Protect the Public’s Health**		
Manage communicable diseases of public health importance	90%	100%
Manage a communicable disease outbreak	89%	100%
Manage environmental health risks, exposures and incidents of public health importance	90%	100%
Manage a cluster of cancer or other adverse health outcome	90%	100%
Prepare for and manage public health incidents and emergencies	90%	80%
**Domain: Promote Health and Prevent Diseases and Injuries**		
Conduct a health policy analysis	90%	100%
Lead and manage strategies and programming to promote health and health equity	90%	100%
Advocate for the adoption and implementation of healthy public policies	89%	100%
Design and manage a population-based screening program	100%	100%

* Note: the EPAs shown are the post-survey version, which have minor wording changes in selected items

## Appendix A

**Table:** Example of making explicit distinctions and inter-relations among EPAs

EPA 5 Conduct an Environmental Health Risk Assessment	Relation to Other EPAs
This EPA includes the conduct of an environmental health risk assessment to characterize the nature and magnitude of health risks to humans from chemical contaminants and other stressors that may be present in the environment. This includes: identifying a hazard and assessing exposure characterizing and managing the risk communicating the risk. Example: assess and report on the health impacts of wind turbines	EPA 4 – Conduct a health impact assessment This EPA focuses on assessing health impacts of a broader range of policies and scenarios such as a municipal redevelopment plan, the introduction of a casino in a city/neighbourhood, etc.
	EPA 14 - Manage environmental health risks, exposures, and incidents of public health importance This EPA focuses on managing a scenario involving a potential exposure to a known health risk with particular emphasis on air quality, water safety, and food safety (e.g., a chemical contamination incident of an aquifer supplying community drinking water).

## Appendix B

**Table 4:** Description and four developmental stages of milestones for EPA 13 – “Manage Communicable Diseaess of Public Health Importance”

Description	Transition to Discipline	Foundations of Discipline	Core of Discipline	Transition to Practice
This EPA includes public health management of all aspects of outbreak investigations of reportable diseases including: declaring an outbreak planning and implementing the investigation implementing control measures making recommendations on case management managing internal and external communications coordinating and managing external assistance providing final outbreak reports. ***Relation to Other EPAs:*** This EPA builds upon EPA 12 which focuses on the management of individual cases of diseases. This EPA may escalate into EPA 16 if the outbreak becomes a public health emergency.	Clinically recognize, investigate and manage infectious diseases of public health importance Know the defining characteristics of an outbreak and how to recognize one when it occurs Describe the powers delegated to the Medical Officer of Health within the Public Health Act specific to the management and control of a suspect reportable communicable disease Report cases of notifiable diseases, conditions, and unusual diseases or patterns to public health authorities Coordinate management of individuals and families with broader public health investigation (e.g., patient education, contact tracing, immunization, treatment.	Describe the steps in an outbreak investigation Establish a case definition for case finding, hypothesis generation and testing in an outbreak scenario/simulation Provide a descriptive analysis (time, place and person) of cases in an outbreak scenario/simulation Conduct the case control or cohort study hypothesis testing analysis in an outbreak scenario/simulation.	Assume under *direct supervision* support roles for managing a real or simulated communicable disease outbreak including: Determining the population at risk Develop a plan for case finding Generate a hypothesis Validate a hypothesis against established facts Develop a case control/cohort study to test a hypothesis Develop the laboratory investigation plan Develop the communication strategy for internal and external audiences Produce the findings and lessons learned report.	Assume under *minimal supervision* the lead role for managing a real or simulated communicable disease outbreak including: Assess available information to decide whether there is an outbreak Declare an outbreak Organize an outbreak team Plan and implement the outbreak investigation including: ○ Investigations to be undertaken (Epidemiologic; Analytic; Laboratory) ○ Standardizing and coordinating sample collection ○ Coordinating environmental inspections Manage the implementation and monitoring of the control measures (e.g., exclusions, closures) to be used Make recommendations on case management Manage the implementation of a communication strategy including responding to media requests Coordinate and manage external assistance Provide the final outbreak reports Assignment of more junior residents to support roles.
